# Impacted third molars and their influence on second molar pathologies: radiological patterns, statistical analysis, and emerging AI perspectives

**DOI:** 10.3389/fdmed.2026.1812621

**Published:** 2026-05-22

**Authors:** Ruxandra-Elena Luca, Mona-Alexandra Cica, Ioana-Roxana Munteanu, Eduard Gidea-Paraschivescu, George-Dumitru Constantin, Mariana-Ioana Miron

**Affiliations:** 1 University Clinic of Oral Rehabilitation and Dental Emergencies, Faculty of Dentistry, “Victor Babes” University of Medicine and Pharmacy, Timisoara, Romania; 2 Interdisciplinary Research Center for Dental Medical Research, Lasers and Innovative Technologies, Timisoara, Romania; 3 Young Graduate, Faculty of Dentistry, “Victor Babes” University of Medicine and Pharmacy, Timisoara, Romania; 4 Oral and Maxillofacial Surgery Clinic, Emergency University Municipal Hospital Timisoara, Timisoara, Romania; 5 Discipline of Clinical Practical Skills, Department I Nursing, Faculty of Medicine, Victor Babeș University of Medicine and Pharmacy, Timișoara, Romania; 6 Doctoral School, “Victor Babes” University of Medicine and Pharmacy Timisoara, Timisoara, Romania

**Keywords:** artificial intelligence (AI), dental radiography, impacted third molars, machine learning, second molar

## Abstract

**Background/objectives:**

Impacted mandibular third molars (M3) often contribute to pathologies of adjacent second molars (M2), including periodontal disease, infections, and caries. This study evaluated how the position of impacted lower wisdom teeth influences M2 pathologies and explored the potential of AI-assisted radiographic analysis.

**Methods:**

A retrospective review of 134 orthopantomograms with impacted or semi-impacted M3s was conducted to assess disease prevalence in both M2 and M3. AI-based image processing tools were applied experimentally to evaluate diagnostic accuracy and limitations.

**Results:**

The findings are organized into three primary sections: the distribution of healthy and diseased teeth for each of the two molars, the types of diseases detected in the second molar, and the overall frequency of pathologies associated with both the second molar and the wisdom tooth. Distal caries of M2 were the most common pathology (59.7%), followed by periodontal bone loss (32.1%) and external root resorption (11.9%). Coronal caries of M3 were observed in 47.0% of cases. Mesioangular and deeper impactions showed the strongest associations with M2 disease. Artificial Intelligence(AI)-assisted image analysis tools were utilized in this study to support the evaluation of dental radiographs.

**Conclusions:**

Distal caries and periodontal involvement are mostly determined by impaction type and depth, particularly mesioangular and deeper impactions (Pell & Gregory IIB, IIIB). The finding that M2 distal caries is the most common pathology, affecting nearly 60% of cases, and that M2 caries significantly increase the likelihood of M3 caries reinforces the idea that identifying high-risk patterns, such as mesioangular and deeper impactions, enables clinicians to anticipate associated pathologies and customise management strategies effectively. While current AI tools face challenges in precise dental diagnosis, their integration shows promise for improving radiographic interpretation and supporting clinical decision-making.

## 1 Introduction

The mandibular third molar (M3), or lower wisdom tooth, is the most distal tooth in the dental arch and exhibits significant morphological variability. It may have four cusps similar to the lower second molar or five rounded cusps resembling the first molar, while the occlusal surface can present smaller enamel protrusions or additional tubercles such as those of Bolk, sextum, or intermedium. Most M3s possess two roots that may fuse or diverge in various directions, with corresponding variations in pulp chamber and root canal morphology (1). M3 formation begins between 7 and 9 years of age, followed by crown calcification from 9 to 14 years and root formation thereafter. Eruption typically occurs between 18 and 25 years, although timing and morphology vary with genetic and environmental influences. Panoramic radiographs remain a valuable tool for assessing M3 development, with average completion ages of 22.36 ± 2.81 years in males and 21.86 ± 2.62 years in females (2, 3).

Due to its anatomical position and variable eruption, M3 is frequently associated with dental complications such as crowding, caries, pericoronaritis, root resorption, cysts, and, rarely, neoplastic transformation (4, 5). The incidence of impaction varies widely, from 16.7% to 68.6%, with no consistent sex predilection, though some studies report slightly higher rates in women (6). Impaction most often results from delayed tooth maturation and inadequate arch space, but may also be influenced by skeletal growth restriction, abnormal tooth angulation, dense alveolar bone, cysts, infections, or systemic conditions (7).

The proximity of impacted M3s to second molars (M2s) often leads to distal caries, root resorption, and periodontal disease of the adjacent tooth. Distal caries, reported in up to 42% of cases, develop in plaque-retentive areas created by mesioangular or horizontally impacted M3s that contact the cemento-enamel junction of M2 (8).

The continuous pressure from the impacted M3 on the M2 may lead to root resorption of the latter. External root resorbtion (ERR) primarily arises from the mechanical pressures exerted by the impacted M3 during its eruption attempt. These forces apply pressure on the root of the M2, resulting in inflammatory resorption of the M2. The proximity of the affected teeth to the M2 may impede the blood arteries of the M2, leading to inflammation and resorption (9). This resorption may undermine the integrity of the M2 and compromise its long-term stability. The impaction of the M3 may cause periodontal complications in the M2 through several mechanisms. The impacted position of the M3 favors the accumulation of bacterial plaque between the M3 and M2, leading to gingival inflammation and the formation of periodontal pockets. Chronic inflammation leads to the development of periodontal pockets between the teeth, where bacteria can harm the supporting tissues. Chronic inflammation and infection result in the degradation of the alveolar bone surrounding the second molar, leading to diminished bone support and increased mobility of the second molar. These periodontal processes can lead to loss of stability and eventual loss of the second molar if not managed appropriately (3). The risk of plaque accumulation and periodontal pathology is primarily associated with partially erupted or mesioangular impactions, rather than fully bone-impacted M3.

Given these potential complications, accurate radiographic assessment of M3 position and development is essential. Orthopantomography remains the standard imaging modality, now increasingly enhanced by artificial intelligence (AI)–based analysis. The detection and characterisation of maladies is one of the most significant effects of AI in medical radiography. AI systems, particularly those using deep learning and convolutional neural networks (CNNs), have demonstrated strong performance in medical image interpretation, often comparable to expert radiologists (8–11). Their ability to identify pathologies rapidly supports early diagnosis, prognosis, and treatment planning, reflecting a shift toward data-driven, patient-centered care (12, 13).

In dentistry, AI has shown promise in detecting impacted teeth, predicting complications, and optimizing pre- and postoperative planning (14–21). The recent development of generative models, including large language models (LLMs) such as the GPT series, has expanded AI's capability to interpret and communicate clinical findings through text-based analysis (13, 22).

Consequently, the aim of our research is to examine the possible complications that M3 may create at the site of mandibular M2, utilizing traditional methods (such as orthopanotomography analysis) alongside artificial intelligence-supported tools, in order to gain a contemporary perspective on a well-established issue in dentistry. The rationale for assessing integration of AI tools, within the framework of dental panoramic x-ray analysis is multifaceted. The unique challenges of dental radiography, such as interpreting nuanced imagery and requiring high diagnostic precision, present optimal conditions for evaluating their adaptability and effectiveness. The potential for different AI-tools to enhance dental diagnostic processes through rapid and accurate analysis of x-rays may significantly improve patient care by facilitating earlier detection of conditions and enabling timely interventions. This research aligns with the objective of integrating AI into healthcare systems to improve service delivery and patient outcomes.

This study originates from the common clinical observation that impacted wisdom teeth may lead to the formation of lesions, including distal caries, periodontal disease, or even root resorption of the second molar, often without evident symptoms in the patient. The investigation is descriptive and observational, and it is predicated on the examination of available radiological images that meet specific criteria. Therefore, the main directions pursued in our study were:

- Position of the lower wisdom molar, as assessed by the following: Winter classification (vertical, mesial-angulated, disto-angulated, horizontal, etc.); Pell & Gregory classification (class I, II, III/A, B, C);
- Evaluation of the relationship between M3 impaction characteristics and M2 pathology
- Assessment of pathology distribution: patient gender (M/F); age group (18–30, 31–45, 46–60 years); presence of associated dental pathologies, including distal caries at the M2 level; signs of periodontal damage at the M2 level (evaluated radiologically by distal bone loss); external root resorption at the M2 level; coronal caries at the M3 level.
- Exploratory use of AI-assisted radiographic tools

## 2 Materials and methods

The study was conducted in accordance with a well-defined protocol that encompassed the collection and analysis of clinical and radiological data and under the approval of the Ethics Committee from the Victor Babes University of Medicine and Pharmacy, Timisoara (No. 52/06.03.2025).

This study was conducted and reported in accordance with the STROBE (Strengthening the Reporting of Observational Studies in Epidemiology) guidelines for retrospective observational research. While the primary focus was exploratory evaluation of AI-assisted dental radiograph interpretation, relevant aspects of TRIPOD (Transparent Reporting of a Multivariable Prediction Model for Individual Prognosis or Diagnosis) were also considered to ensure transparency in describing the AI component.

### 2.1 Data collection

The investigation concentrated on the examination of the correlation between the position of the M3 and M2 pathologies, with a reference sample that was selected in accordance with well-defined criteria. Patients with impacted or semi-impacted M3 and the neighboring M2 present on the arch comprise the target population of this study. The following inclusion criteria were used to identify the 134 orthopantomographies that comprised the study sample:

- The orthopantomographies of patients with impacted or semi-impacted M3 used in this study were obtained from the institutional image archive of the University Clinic of Oral Rehabilitation and Dental Emergencies, Faculty of Dentistry, “Victor Babes” University of Medicine and Pharmacy, Timisoara, Romania. The radiographs belong to patients treated in the university clinic over the past five years and were originally collected for educational and clinical documentation purposes. All images were fully anonymized prior to analysis, and their use was approved in accordance with institutional ethical standards.
- Clear radiographic visibility of the M3 and the neighbouring M2, particularly at the distal surface of M2.
- Clear visualizations of the root structure, tooth positioning, and bone level of the third molars
- Absence of generalized caries or advanced periodontal disease
- OPGs were excluded from the study due to the absence of M2 adjacent to impacted M3, which were used for the classifications and analyze.

The sample selection was conducted in a manner that would guarantee data homogeneity and facilitate an objective examination of the factors that contribute to the development of M2 pathologies.

The analysis was limited to panoramic images (OPG) that enabled a distinct visualisation of the lower wisdom molar and its relationship with the second molar under radiographic conditions that were suitable for evaluation. A total of over 200 orthopantomograms were initially screened; however, only 134 met the inclusion criteria after excluding radiographs with inadequate image quality, incomplete anatomical visualization, or missing clinical data.

The following data were obtained by a singular, calibrated examiner and categorized for each included radiograph:

– patient gender (M/F);
– age group (18–30, 31–45, 46–60 years);
– position of the lower wisdom molar, as assessed by the following:
  - Winter classification (vertical, mesial-angulated, disto-angulated, horizontal, etc.);
  - Pell & Gregory classification (class I, II, III/A, B, C);—presence of associated dental pathologies, including distal caries at the M2 level;
  - signs of periodontal damage at the M2 level (evaluated radiologically by distal bone loss);
  - external root resorption at the M2 level;
  - coronal caries at the M3 level.

### 2.2 Data statistical analyze

All data were introduced into Microsoft Excel (Microsoft Corp., Redmond, WA, USA) and furthermore analyzed using the Statistics Kingdom online platform (https://www.statskingdom.com/). On this platform, the data undergoes statistical tests (Chi-square, Fisher's exact, and Odds Ratio), which produce results as good as with standard statistical packages. To ensure the methodological reproducibility of the statistical analysis, the same statistical data (*p*-values, *χ*², and confidence intervals) were double-checked using JASP version 0.95 (University of Amsterdam, The Netherlands), an open-source statistical software based on the R environment. The same numerical values obtained with both statistical tools confirm the reliability of Statistics Kingdom platform.

Descriptive statistics were expressed as absolute frequencies (n) and percentages (%). Chi-square or Fisher's exact test, with statistical significance defined as *p* < 0.05, were used to perform comparisons among subgroups (sex, age and impaction type).

A *post hoc* power analysis for the main Chi-square comparison (α = 0.05, w = 0.3, df = 2, *n* = 134) yielded an achieved power of 0.81, confirming that the sample size was sufficient to detect moderate effects. Smaller categories (e.g., Pell & Gregory IIC and IIIC) were analyzed descriptively due to low counts.

Prevalence values were calculated separately for the overall cohort as well as for each subgroup of interest (sex, age category, Winter classification, Pell & Gregory classification). For each subgroup j, prevalence was defined as the proportion of positive cases (xj) relative to the total number of subjects in that subgroup (nj):

$$Prevalence_{X|j}=\frac{x_{j}}{n_{j}}\times 100\text{\%}$$

When reporting by sex or age, patients with missing demographic information were excluded from the denominator, and the effective group size (n) was explicitly specified.

In addition to prevalence within subgroups, the share of positives across subgroups was also computed, defined as the proportion of cases within a given subgroup relative to the total number of positive cases across the entire cohort:

$$Prevalence_{X|j}=\frac{x_{j}}{n_{j}}\times 100\text{\%}$$

Comparisons of prevalence between subgroups were performed using the Chi-square (*χ*²) test of independence. When expected frequencies in any contingency table cell were <5, Fisher's exact test was applied to ensure validity. Statistical significance was defined as *p* < 0.05 (statistically significant).

All percentages were reported to one decimal place. For categories with very small sample sizes (e.g., Pell & Gregory IB, IIC, IIIC), both absolute counts and percentages were provided (e.g., 3/3 = 100%) to maintain transparency. Results from these categories were interpreted with caution due to limited statistical power.

### 2.3 Emerging AI tools perspectives

The secondary section of the study included the selection of several representative radiographs, which were analysed using the current AI software available on the market (ChatGPT-5:OpenAI, Inc. San Francisco, CA, U.S.A., x-ray Interpreter: RadAI- SanFrancisco, CA, U.S.A., Diagnocat:DGNCT LLC, Miami, Florida, U.S.A.).

AI-assisted image analysis tools were utilized in this study to support the evaluation of dental radiographs. These systems apply advanced machine learning algorithms to identify and annotate anatomical structures and potential pathological findings, thereby facilitating a more standardized and objective assessment process. The incorporation of AI in dental radiographic analysis within this research is exploratory in nature, intended to examine the technology's diagnostic potential, reliability, and limitations. The approach aims to complement, rather than substitute, expert clinical judgment, providing a basis for future validation and integration of AI methodologies in dental diagnostic practice.

The AI model was trained utilising comprehensive dental education materials, incorporating training with visual aids, a numbering system, and instructions for disregarding non-relevant areas. The protocol is described in detail, below. All images were fully anonymized prior to processing, ensuring that no patient-identifiable information was accessed or used, in accordance with institutional and regulatory standards.

#### 2.3.1 ChatGPT-5

Initially, the radiograph was uploaded, and a request was formulated to ChatGPT-5 to locate on the dental orthopantomograph (Figure 1) the impacted mandibular molar on the patient's right side (the left side of the radiograph) and provide the appropriate impaction classification based on the Pell & Gregory and Winter classifications. We also required information regarding the presence of any pathologies in the adjacent second molar, including caries, bone loss, or other dental issues.

**Figure 1:**
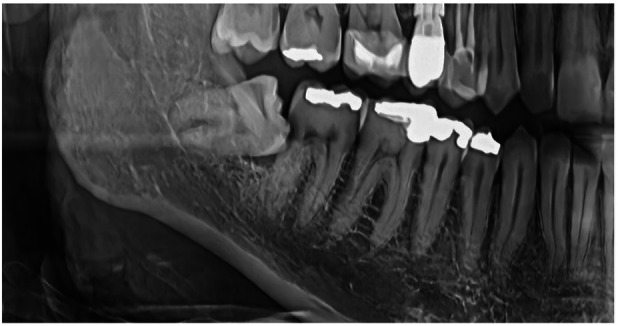
The OPG aspect of a right mandibular third molar impaction, along with the secondary carious lesion and periodontal involvement on the distal surface of the adjacent second molar (cropped size).

The initial information from the chatbot and the schematic representation were inadequate (Figure 2), failing to identify and to localize the area of interest, prompting us to proceed to the second stage, where we supplied additional information to elicit a more precise response.

**Figure 2:**
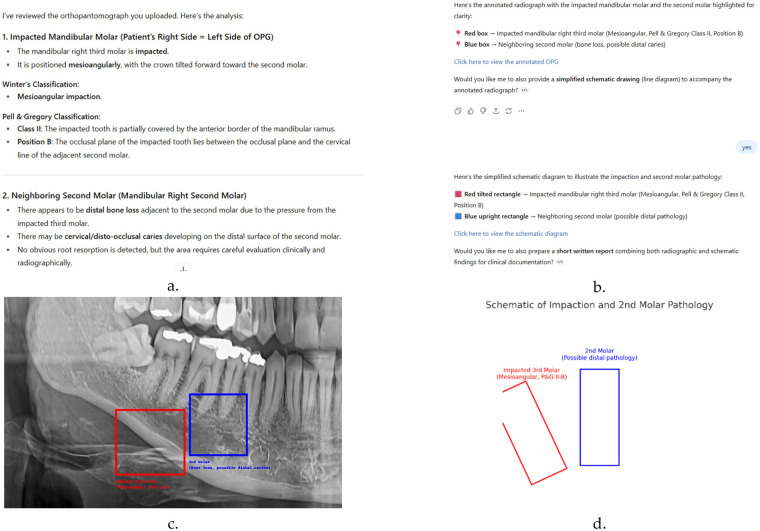
The initial response from the chatbot **(a,b)**, along with the incorrect identification of areas of interest **(c)** and the flawed graphic representation **(d)**.

The teeth were marked on the OPG, with the included M3 designated as 4.8 and the adjacent M2 as 4.7. and requested a re-evaluation of the analysis.

This time, with the addition of new guiding information, the interpretation is more accurate (Figure 3).

**Figure 3:**
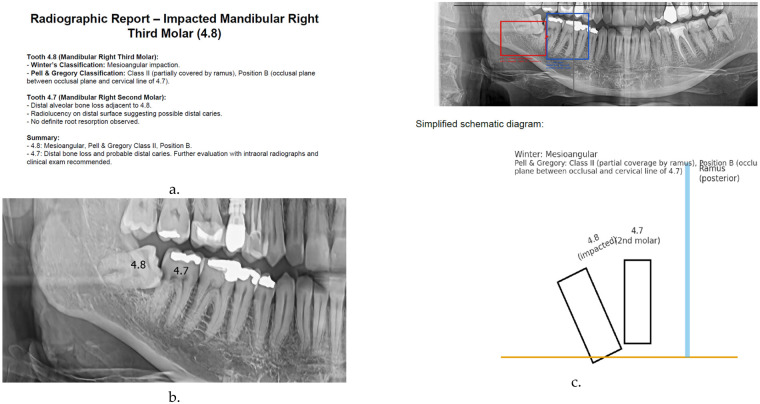
The second interpretation from the chatbot **(a)**, along with annotated radiograph **(b)**, the correct identification of areas of interest and the second graphical representation **(c)**.

Although under proper guidance, ChatGPT-5 managed to achieve more satisfactory results, it is advisable to be incorporated as a complementary AI component to facilitate text-based synthesis and evaluation of diagnostic information derived from image dedicated analysis tools.

#### 2.3.2 X-ray interpreter

A comparison was conducted using identical data provided to the x-Ray Interpreter software, resulting in the analysis presented below as an example (Figure 4). In this scenario, unlike the previous case, interactive communication with a chatbot is not feasible; thus, the radiograph analysis report is produced automatically.

**Figure 4:**
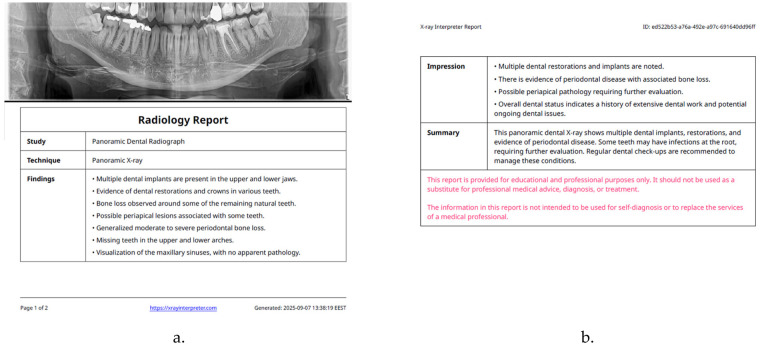
X-Ray interpreter report obtained via https://xrayinterpreter.com/ **(a,b)**, illustration on the identical radiograph.

#### 2.3.3 Diagnocat

The third AI tool employed is specifically calibrated for dental radiology, resulting in an analysis that is significantly more detailed and accurate compared to the previous two tools (Figure 5).

**Figure 5:**
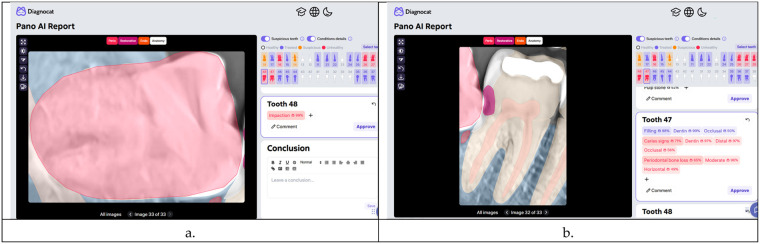
Diagnocat interpretation of the the provided panoramic x-ray, emphasising the impaction status of the third molar **(a)**, along with the periodontal and dental issues concerning the adjacent second molar **(b)**.

The results are detailed in the corresponding section.

The artificial-intelligence component evaluation was exploratory and qualitative, designed to compare interpretive performance across different commercial platforms. As no standardized ground-truth dataset was available, diagnostic accuracy metrics (sensitivity, specificity, Cohen's κ) were not computed. These indicators will be incorporated in a subsequent prospective validation study using annotated radiographs.

The findings were intentionally presented in a structured manner by separately reporting the distribution of pathologies affecting the second molar (M2), the third molar (M3), and their combined patterns. This approach was adopted to enhance clarity and allow a more precise characterization of disease distribution for each tooth, while also facilitating a structured interpretation of the clinically relevant relationships between them. By distinguishing these components, the analysis provides a clearer understanding of how impacted third molars may influence adjacent second molar pathology. The rationale for this reporting structure has been clarified in the manuscript to improve coherence and readability.

## 3 Results

This section presents the outcomes of both descriptive and inferential analyses performed on the study cohort. The results are structured according to key clinical and radiological domains, namely the overall prevalence of pathologies, subgroup distributions by sex and age, and associations with positional classifications (Winter, Pell & Gregory). Where applicable, inferential statistics (*χ*² or Fisher's exact tests) are reported alongside descriptive findings to evaluate the statistical significance of observed differences.

### 3.1 Overall prevalence of pathologies

From the analysis of 134 panoramic radiographs, distal caries of the M2 emerged as the most frequent pathology, affecting 80 patients (59.7%). Periodontal involvement of M2, defined as radiographic evidence of distal bone loss, was present in 43 cases (32.1%), while external root resorption of M2 was observed in 16 cases (11.9%). At the level of the M3, coronal caries were identified in 63 cases (47.0%).

It is important to note that these percentages are not mutually exclusive, since many x-rays revealed multiple concomitant conditions. The values should therefore be interpreted as individual prevalence rates for each pathology, rather than as parts of a single whole.

The prevalence of caries and periodontal changes in M2 was notably higher in patients with mesioangular impactions, suggesting a positional influence on disease susceptibility.

To provide a comprehensive overview, Figure 6 presents the overall prevalence of each pathology as a bar chart, illustrating the relative frequency of distal caries, periodontal involvement, root resorption, and third molar caries across the full cohort.

**Figure 6:**
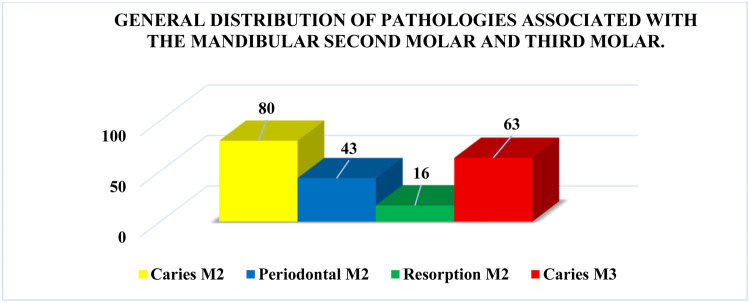
Overall prevalence of pathologies associated with the M2 and M3. Values are expressed as percentages of the total study population (*n* = 134). Each condition was assessed independently, as individual patients could present with more than one pathology.

### 3.2 Distribution by quadrant (III vs. IV)

The analysis of quadrant-specific distribution revealed moderate differences be-tween the left mandibular quadrant (III) and the right mandibular quadrant (IV). In quadrant III, distal caries at the M2 were present in 40 cases (56.3%), periodontal involvement in 20 cases (28.2%), external root resorption in 6 cases (8.5%), and caries at the M3 in 31 cases (43.7%). In quadrant IV, frequencies were slightly higher: distal caries at M2 were diagnosed in 40 cases (63.5%), periodontal involvement in 23 cases (36.5%), root resorption in 10 cases (15.9%), and M3 caries in 32 cases (50.8%) (Table 1).

**Table 1:** Distribution of mandibular pathologies by quadrant (III vs. IV).

Pathology	Quadrant III *n* (%)	Quadrant IV *n* (%)
Caries M2	40 (56.3%)	40 (63.5%)
Periodontal M2	20 (28.2%)	23 (36.5%)
Resorption M2	6 (8.5%)	10 (15.9%)
Caries M3	31 (43.7%)	32 (50.8%)

Although the overall distribution of pathologies was broadly comparable between the two quadrants, the incidence of periodontal involvement and root resorption was higher on the right side (quadrant IV).To better visualize the asymmetries between the left and right mandibular quadrants, we constructed a comparative bar chart (Figure 7). This figure highlights the moderate differences in the prevalence of each pathology, with IV^th^ quadrant showing slightly higher periodontal involvement and root resorption compared to quadrant III.

**Figure 7:**
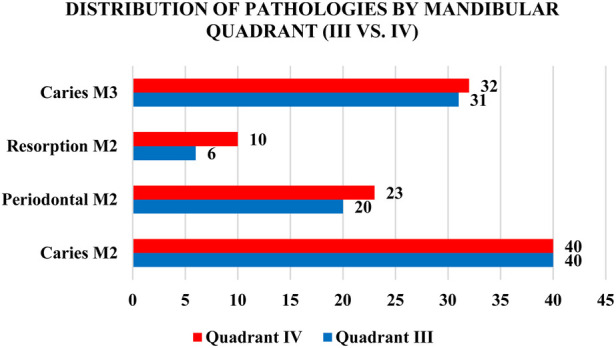
Distribution of pathologies affecting the mandibular second molar (M2) and third molar (M3) according to quadrant (III vs. IV).

When comparing the distribution of pathologies between mandibular quadrants, no statistically significant differences were observed. Distal caries at the M2 were present in 56.3% of cases in III^rd^ quadrantand 63.5% in IV^th^ quadrant [*χ*²(1) = 0.44, *p* = 0.505]. Periodontal involvement of M2 was slightly more frequent in IV^th^ quadrant (36.5%) compared with III^rd^ quadrant (28.2%), yet the difference was not significant [*χ*²(1) = 0.72, *p* = 0.397]. Similarly, root resorption of M2 was identified in 8.5% of III^rd^ quadrant cases and 15.9% of IV^th^ quadrant cases, without statistical significance [*χ*²(1) = 1.11, *p* = 0.291]. Caries affecting the M3 were also comparable between quadrants [43.7% vs. 50.8%; *χ*²(1) = 0.43, *p* = 0.514]. These findings suggest that, although IV^th^ quadrant exhibited a tendency toward higher prevalence of periodontal and resorptive changes, the overall distribution of pathologies was balanced between left and right mandibular sides.

### 3.3 Correlation between sex and pathologies

A comparative analysis between female and male patients revealed modest differences in the prevalence of pathologies associated with the M2 and M3. In female patients (*n* = 68), the prevalence of distal caries at M2 was 67.6%, followed by caries at M3 (54.4%), distal periodontal involvement at M2 (33.8%), and external root resorption (13.2%). In male patients (*n* = 57), distal caries at M2 were present in 57.9% of cases, M3 caries in 38.6%, periodontal involvement in 28.1%, and root resorption in 12.3%.

These distributions indicate that women had a higher frequency of both distal caries at M2 and coronal caries at M3, while men showed slightly lower rates for these pathologies but comparable frequencies for periodontal involvement and root resorption. Table 2 summarizes the absolute distribution of cases, and Figure 8 provides a visual comparison.

**Table 2:** Number of cases with pathologies associated with the mandibular second molar (M2) and third molar (M3), stratified by sex.

Sex	Caries M2	Periodontal M2	Resorption M2	Caries M3
F	46	23	9	37
M	33	16	7	22

**Figure 8:**
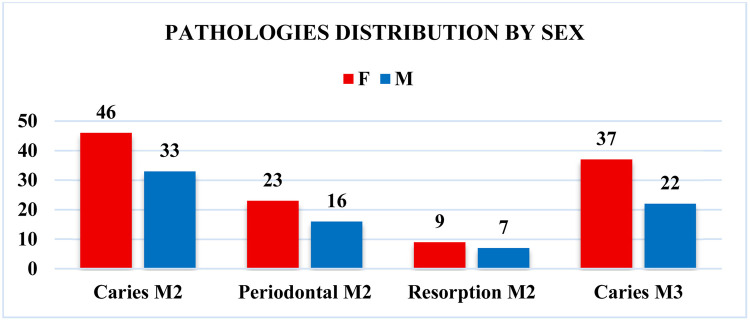
Distribution of mandibular pathologies (M2 and M3) according to sex. Bars represent the absolute number of cases identified in female (red) and male (blue) patients (*n* = 125; cases with missing sex information were excluded).

From an inferential perspective, Chi-square tests did not identify statistically significant differences between sexes for any of the examined pathologies: distal caries at M2 [*χ*²(1) = 1.34, *p* = 0.247], caries at M3 [*χ*²(1) = 3.14, *p* = 0.076], periodontal involvement at M2 [*χ*²(1) = 0.49, *p* = 0.484], and root resorption at M2 [*χ*²(1) = 0.02, *p* = 0.884]. Nevertheless, a clinical trend was observed, with women showing higher rates of caries in both M2 and M3.

### 3.4 Age-related distribution of pathologies

The distribution of pathologies associated with M2 and M3 showed relevant variations across age categories (Table 3, Figure 9,10).

**Table 3:** Distribution of mandibular pathologies by age group.

Age group	Caries M2	Periodontal M2	Resorption M2	Caries M3	Patients
18–30	44	23	6	30	70
31–45	29	13	10	24	46
46–60	6	3	0	5	9

**Figure 9:**
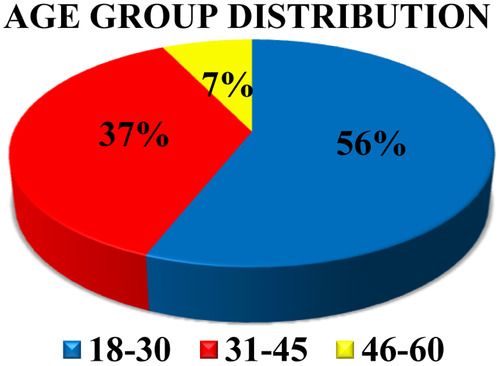
Distribution of patients by age group. The chart illustrates the proportion of patients in the age categories 18–30 years (*n* = 70; 56%), 31–45 years (*n* = 46; 37%), and 46–60 years (*n* = 9; 7%), excluding cases with missing age data (*n* = 125).

**Figure 10:**
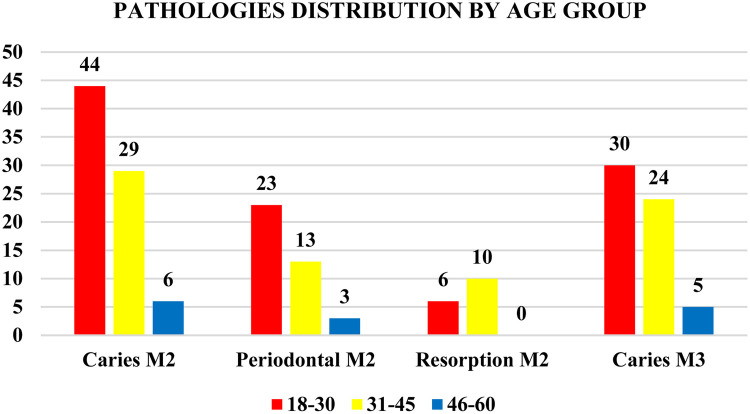
Distribution of pathologies affecting M2 and M3 according to age group. The bar chart displays the absolute number of cases for each pathology across the three age categories (18–30, 31–45, and 46–60 years), highlighting differences in prevalence patterns.

In the 18–30 years group (*n* = 70), distal caries at M2 were most frequent (44 cases, 62.9%), followed by caries at M3 (30 cases, 42.9%). Periodontal involvement was identified in 23 patients (32.9%), while external root resorption was relatively uncommon (6 cases, 8.6%).

In the 31–45 years group (*n* = 46), distal caries at M2 remained highly prevalent (29 cases, 63.0%), and M3 caries were even more frequent than in the younger group (24 cases, 52.2%). Periodontal involvement was reported in 13 cases (28.3%), while root resorption was substantially higher than in the 18–30 group (10 cases, 21.7%).

In the 46–60 years group (*n* = 9), the prevalence of all pathologies was reduced, though still notable in relative terms: 6 cases of M2 caries (66.7%), 5 cases of M3 caries (55.6%), 3 cases of periodontal involvement (33.3%), and no cases of root resorption.

To illustrate these differences, Figure 9 depicts the age distribution of the study cohort, while Figure 10 provides a comparative bar chart of pathological prevalence across age groups.

When tested statistically, differences across age categories did not reach significance for distal caries at M2 [*χ*²(2) = 0.04, *p* = 0.978], periodontal involvement [*χ*²(2) = 0.26, *p* = 0.879], or caries at M3 [*χ*²(2) = 1.16, *p* = 0.559]. However, external root resorption demonstrated a clear age trend, being more frequent in the 31–45 group compared to both younger and older patients, though the difference narrowly missed statistical significance [*χ*²(2) = 4.28, *p* = 0.118].

### 3.5 Distribution of pathologies according to winter classification

In the analysis of pathologies associated with impacted M3, a clear relationship was observed between Winter's classification of impaction and the occurrence of dental conditions. Figure 11 illustrates the prevalence of the main pathologies (distal caries of M2, periodontal involvement of M2, external root resorption, and caries of M3) across the three most frequent impaction types: mesioangular, distoangular, and vertical.

**Figure 11:**
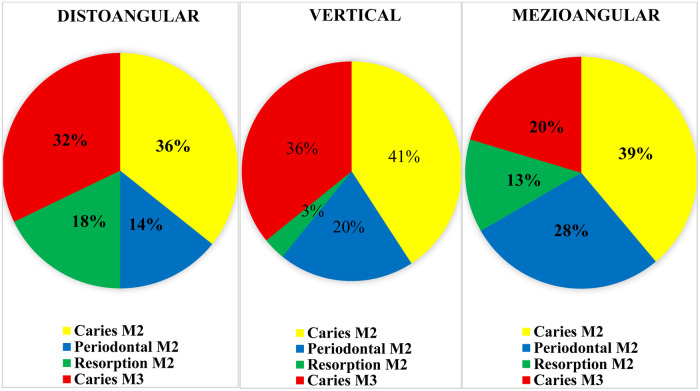
Prevalence of pathologies associated with impacted M3 according to winter's classification (mesioangular, distoangular, vertical). Values are expressed as percentages of cases within each positional category.

The mesioangular position (*n* = 31) was most strongly associated with distal caries at the M2 (67.7%), followed by periodontal involvement (48.4%), caries at M3 (35.5%), and external root resorption (22.6%). These findings confirm that mesioangular impactions represent a major risk factor for multiple pathologies, likely due to reduced interdental space and limited accessibility for oral hygiene.

The distoangular position (*n* = 16) was also strongly associated with pathology, with distal caries at M2 in 62.5% of cases, caries at M3 in 56.2%, external root resorption in 31.2%, and periodontal involvement in 25.0%. Although less common, this orientation appears to favor abnormal contact between M3 and M2, increasing the risk of caries and resorptive changes.

The vertical position (*n* = 87), the most frequently observed type, was associated with lower prevalence of periodontal and resorptive changes. Distal caries at M2 were present in 56.3% of cases, caries at M3 in 49.4%, periodontal involvement in 27.6%, and root resorption in only 4.6%.

Statistical analysis (*χ*² tests) confirmed that the distribution of periodontal involvement [*χ*²(2) = 8.42, *p* = 0.015] and root resorption [*χ*²(2) = 14.21, *p* < 0.001] differed significantly between impaction types, while differences in distal caries at M2 (*p* = 0.423) and caries at M3 (*p* = 0.732) were not statistically significant. These results underscore the clinical importance of mesioangular and distoangular positions as higher-risk orientations.

### 3.6 Distribution of pathologies according to pell & gregory classification

Marked differences in pathology prevalence were also observed across the Pell & Gregory classes (Table 2, Figure 12). In Class IA (*n* = 48), distal caries at M2 were recorded in 64.6% of cases, while caries at M3 were present in 60.4%. Periodontal involvement and root resorption were less frequent (14.6% and 8.3%, respectively).

**Figure 12:**
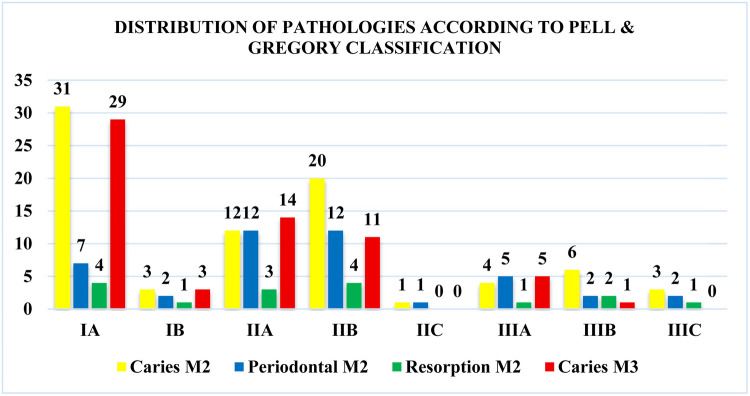
Prevalence of pathologies associated with impacted M3 according to pell & gregory classification (IA–IIIC). Bars represent the percentage of cases affected by distal caries, periodontal involvement, root resorption, and caries at M3 in each positional class.

Class IB (*n* = 3), although very small, demonstrated extremely high values: all cases (100%) presented with caries in both M2 and M3, with 66.7% showing periodontal involvement and 33.3% root resorption.

In Class IIA (*n* = 30), both distal caries at M2 and periodontal involvement were observed in 40.0% of cases, while caries at M3 occurred in 46.7% and root resorption in 10.0%.

Class IIB (*n* = 28) showed the highest prevalence of distal caries at M2 (71.4%), with concomitant periodontal involvement in 42.9%, caries at M3 in 39.3%, and root resorption in 14.3%.

Classes IIIA (*n* = 13), IIIB (*n* = 7), and IIIC (*n* = 3) displayed variable patterns. In IIIA, caries at M2 were less frequent (30.8%), while periodontal involvement and caries at M3 occurred in 38.5%. In IIIB, distal caries at M2 were dominant (85.7%), while M3 caries were rare (14.3%). In IIIC, all patients (100%) presented with M2 caries, with two-thirds showing periodontal involvement and one-third root resorption.

Chi-square testing indicated statistically significant differences in the distribution of periodontal involvement [*χ*²(7) = 15.63, *p* = 0.028] and root resorption [*χ*²(7) = 14.87, *p* = 0.037] across Pell & Gregory classes, while differences for M2 and M3 caries did not reach statistical significance (*p* > 0.05).

### 3.7 Co-occurrence of pathologies

To investigate whether pathologies of the M2 and M3 tend to co-occur, a cross-tabulation of cases was performed (Table 4). Distal caries of M2 were most frequently associated with other conditions: in 26 cases (20.8%) they co-occurred with periodontal involvement, in 12 cases (9.6%) with root resorption, and in 49 cases (39.2%) with M3 caries. Similarly, M3 caries were found concomitantly with periodontal changes in 17 cases and with root resorption in 6 cases. The least frequent association was between periodontal involvement and root resorption (8 cases, 6.4%). Table 5 presents a heatmap of the co-occurrence of pathologies in M2 and M3.

**Table 4:** Prevalence of mandibular pathologies according to pell & gregory classification. Values are reported as absolute numbers and percentages of cases within each class.

Class	N	Caries M2		Periodontal M2		Resorption M2		Caries M3	
		*N*	%	*N*	%	*N*	%	*N*	%
IA	48	31	64.6	7	14.6	4	8.3	29	60.4
IB	3	3	100	2	66.7	1	33.3	3	100
IIA	30	12	40	12	40	3	10	14	46.7
IIB	28	20	71.4	12	42.9	4	14.3	11	39.3
IIC	2	1	50	1	50	0	0	0	0
IIIA	13	4	30.8	5	38.5	1	7.7	5	38.5
IIIB	7	6	85.7	2	28.6	2	28.6	1	14.3
IIIC	3	3	100	2	66.7	1	33.3	0	0

**Table 5:** Heatmap of co-occurrence of pathologies in the M2 and M3.

Heatmap of pathologies	Caries M2	Periodontal M2	Resorption M2	Caries M3
Caries M2	80	26	12	49
Periodontal M2	26	43	8	17
Resorption M2	12	8	16	6
Caries M3	49	17	6	63

Values are absolute counts; cell shading intensity reflects frequency (white = low frequency, red = high frequency).

From an inferential standpoint, the Chi-square test demonstrated a highly significant association between distal caries at M2 and caries at M3 (*χ*²(1) = 21.84, *p* < 0.001), indicating a strong tendency for these two conditions to appear together. The association between M2 caries and periodontal involvement was also statistically significant (*χ*²(1) = 6.52, *p* = 0.011). Conversely, the co-occurrence of root resorption with other conditions did not reach statistical significance (all *p* > 0.05), consistent with its overall lower prevalence (Table 6).

**Table 6:** Statistical associations between mandibular pathologies (Chi-square tests).

Pathology Pair	*χ*² (df)	*p*-value	Interpretation
Caries M2 × Caries M3	21.84 (1)	<0.001	Strong positive association: patients with distal caries at M2 were significantly more likely to also present with caries at M3.
Caries M2 × Periodontal M2	6.52 (1)	0.011	Significant association: M2 caries tend to co-occur with periodontal involvement.
Caries M2 × Resorption M2	1.77 (1)	0.184	Not significant, though a mild non-significant trend was observed.
Periodontal M2 × Caries M3	1.42 (1)	0.233	No significant association.
Periodontal M2 × Resorption M2	2.67 (1)	0.102	Borderline trend toward association, but not statistically significant.
Resorption M2 × Caries M3	0.66 (1)	0.416	No significant association.

These findings highlight that patients presenting with distal caries at M2 are at substantially greater risk of also exhibiting M3 caries and periodontal deterioration.

### 3.8 Correlation between sex and positional classifications (winter and pell & gregory)

#### 3.8.1 Sex and winter classification

To assess potential sex-related differences in the positional distribution of impacted mandibular third molars, cases were analyzed according to the Winter classification. Out of the total cohort of 134 x-rays, 9 lacked complete classification data, leaving 125 eligible cases. The overall distribution showed that 84 cases (67.2%) were vertical impactions, 28 cases (22.4%) mesioangular, and 13 cases (10.4%) distoangular (Table 7).

**Table 7:** Distribution of cases by winter classification and sex.

Winter Classification	Females (*n* = 68)	Males (*n* = 57)	Total (*n* = 125)
Distoangular	4	9	13
Mesioangular	15	13	28
Vertical	49	35	84
Total	68	57	125

When stratified by sex, the following frequencies were observed: Females (*n* = 68): vertical—49 (72.1%), mesioangular—15 (22.1%), distoangular—4 (5.9%). Males (*n* = 57): vertical—35 (61.4%), mesioangular—13 (22.8%), distoangular—9 (15.8%) (Figure 13).

**Figure 13:**
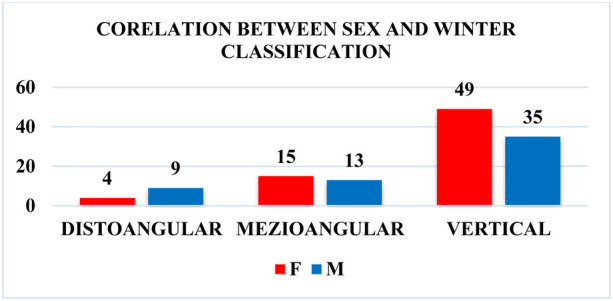
Distribution of M3 positions (winter classification) according to sex.

A Chi-square test of independence did not reveal a statistically significant association between sex and Winter classification [*χ*²(2) = 3.25, *p* = 0.197]. However, a clinical tendency was noted: males more frequently exhibited distoangular impactions, whereas females showed a predominance of vertical positions.

#### 3.8.2 Sex and pell & gregory classification

A similar analysis was performed using the Pell & Gregory classification. From the 134 patients, 125 had complete data. The global distribution was: IA—48 cases (38.4%), IB—3 (2.4%), IIA—30 (24.0%), IIB—28 (22.4%), IIC—2 (1.6%), IIIA—13 (10.4%), IIIB—7 (5.6%), and IIIC—3 (2.4%) (Table 8).

**Table 8:** Distribution of cases by Pell & Gregory classification and sex.

Pell & Gregory Class	Females (*n* = 68)	Males (*n* = 57)	Total (*n* = 125)
IA	32	16	48
IB	1	2	3
IIA	12	17	29
IIB	10	12	22
IIC	0	2	2
IIIA	7	4	11
IIIB	5	2	7
IIIC	1	2	3
**Total**	68	57	125

Sex-stratified distribution showed: Females (*n* = 68): IA—26 (38.2%), IB—1 (1.5%), IIA—18 (26.5%), IIB—15 (22.1%), IIC—1 (1.5%), IIIA—5 (7.4%), IIIB—1 (1.5%), IIIC—1 (1.5%). Males (*n* = 57): IA—22 (38.6%), IB—2 (3.5%), IIA—12 (21.1%), IIB—13 (22.8%), IIC—1 (1.8%), IIIA—8 (14.0%), IIIB—6 (10.5%), IIIC—2 (3.5%) (Figure 14).

**Figure 14:**
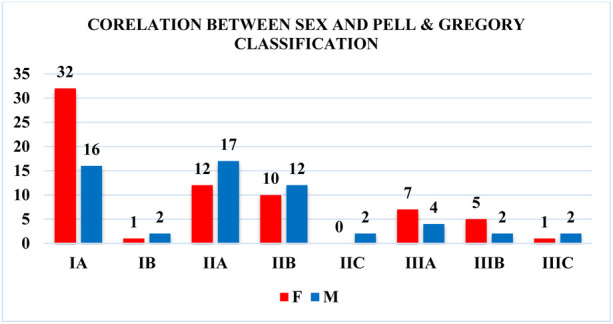
Distribution of mandibular third molar positions (pell & gregory classification) according to sex.

The Chi-square test confirmed no statistically significant differences between sexes for Pell & Gregory classes [*χ*²(7) = 6.48, *p* = 0.486]. Still, females tended to cluster in superficial positions (IA), while males appeared more frequently in deeper positions (IIA, IIIB, IIIC).

### 3.9 Predictive associations between pathologies

To explore potential predictive relationships between pathologies of M2 and M3, odds ratios (OR) with 95% confidence intervals (CI) were calculated from 2 × 2 contingency tables (Table 9). Fisher's exact test was used to assess statistical significance due to the presence of cells with small, expected counts.

**Table 9:** Predictive associations between mandibular pathologies and positional factors (odds ratios, 95% CI, Fisher's exact test).

Predictor	Outcome	OR	95% CI	*p*-value	Interpretation
Caries M2	Caries M3	4.52	2.12–9.63	<0.001	Significant positive association
Caries M2	Periodontal M2	1.05	0.50–2.20	1.000	No significant association
Mesioangular impaction	Resorption M2	6.05	1.63–22.42	0.003	Significant predictor; mesioangular > vertical
Mesioangular impaction	Caries M2	1.63	0.69–3.86	0.266	No significant association
Pell IIB vs. IA	Caries M2	0.73	0.27–2.00	0.540	No significant association

The analysis revealed that distal caries at M2 were a strong predictor of caries at M3. Patients with M2 caries were 4.5 times more likely to also present with caries at M3 compared to those without M2 caries (OR = 4.52; 95% CI: 2.12–9.63; *p* < 0.001). This finding highlights the interdependence of pathological processes in adjacent molars and underscores the cumulative risk of disease progression in the posterior mandibular region.

In contrast, the association between distal caries at M2 and periodontal involvement at M2 was not statistically significant (OR = 1.05; 95% CI: 0.50–2.20; *p* = 1.000), suggesting that these two conditions may occur independently rather than predictively.

Beyond inter-pathological associations, positional predictors were also evaluated. Mesioangular impaction was found to significantly increase the risk of external root resorption of M2 when compared to vertical impaction (OR = 6.05; 95% CI: 1.63–22.42; *p* = 0.003). This confirms mesioangular positioning as a high-risk anatomical factor, predisposing adjacent second molars to root damage through altered contact surfaces and mechanical stress. By contrast, other positional comparisons, such as mesioangular vs. vertical for M2 caries (OR = 1.63; 95% CI: 0.69–3.86; *p* = 0.266), did not reach statistical significance.

## 4 Discussions

This study provides a novel contribution by jointly analyzing impaction characteristics and the spectrum of associated second molar pathologies within a single framework, while also incorporating AI-assisted radiographic analysis as an exploratory approach to evaluate its emerging role in dental diagnostics.

### 4.1 Statistical results and predictive risk factors

This study provides a radiographic evaluation of pathologies associated with impacted third molars (M3) and adjacent second molars (M2) across 134 cases. Key patterns emerged regarding prevalence, co-occurrence, demographic factors, and positional risks.

Distal caries of M2 were the most common finding, affecting nearly 60% of cases, underscoring the susceptibility of M2 in the presence of an impacted M3. Odds ratio analysis showed that M2 caries strongly predicted M3 caries, with affected patients being over four times more likely to have lesions in both teeth—highlighting their pathological interdependence. Periodontal involvement (32%) and external root resorption (12%) were less frequent but clinically relevant. Periodontal deterioration of-ten co-occurred with caries, though statistical testing indicated largely independent development. Root resorption was rarer but peaked in middle-aged patients, likely due to prolonged mechanical contact between adjacent roots.

Pathologies were symmetrically distributed across mandibular quadrants, showing no laterality effect. However, positional classification revealed meaningful differences: mesioangular impactions and Pell & Gregory classes IIB and IIIB were most associated with distal caries and periodontal involvement, whereas distoangular impactions correlated with root resorption. Vertical impactions, though most common, presented lower rates of complex pathology, emphasizing impaction type over laterality as the key determinant of risk.

A 2021 Iranian study similarly found that while complications from impacted mandibular third molars were relatively infrequent, they remained significant. Among 1,600 cases, 12.2% caused M2 distal caries, 15.8% root resorption, and 7.4% pathological lesions; 54.5% had mandibular canal contact. Impaction angulation is a risk factor for distal caries in second molars, whereas altered angulation and increased impaction depth are associated with root resorption of second molars. The reduced distance between the ramus and the distal aspect of the second molar was identified as a risk factor for associated pathological lesions (23).

In our cohort, no significant sex-based differences were observed in pathology prevalence or impaction type. However, women more often exhibited vertical, superficial impactions, whereas men showed deeper, distoangular ones—patterns consistent with previous reports linking mandibular morphology and impaction orientation. Though not statistically significant, these tendencies have clinical implications for predicting extraction difficulty and postoperative complications. These tendencies align with prior literature reporting dimorphic influences of mandibular morphology on third molar eruption paths (24–27). Epidemiological and multicenter studies similarly indicate that men tend to present with deeper, more angulated impactions, which carry higher surgical difficulty and risk of complications, while women show more vertical orientations that may more often warrant prophylactic removal (28–32). Such trends, though not statistically significant in the present sample, provide clinically useful context for anticipating extraction difficulty and potential sequelae.

Co-occurrence analysis revealed that M2 distal caries often accompanied M3 caries and, to a lesser extent, periodontal disease, underscoring the need to view these as interrelated processes rather than isolated conditions.

Finally, our predictive criteria align with Hur et al. (33), identifying six relevant predictors—sex, age, contact point at the cemento-enamel junction, impaction angulation, and Winter's and Pell & Gregory classifications. Their machine learning models demonstrated that these variables can effectively predict pathology in M2, supporting targeted prevention and informed management of impacted M3.

Our data indicate that although sex-related differences were not statistically significant, female patients demonstrated a higher sensitivity to carious lesions, while periodontal and resorptive changes were uniformly distributed between sexes. The prevalence of caries at both M2 and M3 stays continuously elevated throughout all age groups, but root resorption exhibits greater age dependency, peaking in middle-aged individuals. The very low frequencies in the 46–60 age group may indicate a reduced sample size and potential prior extractions within this demographic. The results show that distal caries at M2 could serve as a clinical indicator for complications of M3, whereas mesioangular impaction can substantially forecast periodontal problems. Collectively, they suggest that both pathogenic and anatomical factors contribute to the overall disease burden. This underscores the imperative of personalised risk evaluation: individuals with mesioangular impactions and pre-existing M2 caries should be meticulously followed or evaluated for early management to prevent irreversible complications.

### 4.2 Exploratory evaluation of AI-assisted tools in dental radiological diagnosis

Artificial intelligence (AI) models are increasingly used in clinical medicine to predict disease risk, detect abnormalities, assist diagnosis, and evaluate prognosis. In radiology, deep learning (DL) approaches have largely replaced traditional machine learning due to their superior features and predictive capacity (21, 34, 35).

Celik et al. (2022, Turkey) (36) developed a deep convolutional neural network for identifying impacted third molars (M3) on panoramic radiographs. Among the tested architectures, YOLOv3 achieved the best results (recall = 0.93, precision = 0.88), confirming its reliability for clinical decision support. In 2023, Lei et al. (China) (37) enhanced YOLOv5s using a knowledge distillation framework with YOLOv5x as the teacher model, improving mean average precision by 2.9% while reducing computational load. Similarly, Maruta et al. (Japan) (16) applied automated machine learning with data augmentation to classify M3 impaction, demonstrating that synthetic image generation can effectively enhance model performance.

Zirek et al. (21) used 1,000 dental radiographs to predict Winter's angulation and localize impacted teeth with YOLOv8, achieving excellent detection metrics (precision = 0.972, recall = 0.967, F1 = 0.969). One of the most potent recent iterations of the YOLO family, the YOLOv8 algorithm (You Only Look Once) has been implemented in artificial intelligence applications for the impacted tooth analysis. They conclude that the clinical interface is developed to enhance internship training for students in dental faculties. Moreover, it can be important in the clinical decision-making process by providing angle position data to anticipate the complexity of tooth extractions.

Another 2025 study employed deep learning based on the Pell & Gregory and Winter classifications and the Pederson Difficulty Index, attaining accuracy compara-ble to manual assessment and supporting workflow optimization and time efficiency (38).

Subsequent work from Turkey (2025) introduced the Mandibular Third Molar (m-TM) dataset and trained CNNs with transfer learning, obtaining 87% accuracy, 75% sensitivity, and 95% specificity. Clinical evaluations confirmed its reliability and potential for integration into diagnostic systems (10).

Our study found that AI-based x-ray analysis software did not accurately interpret panoramic x-rays in many situations, highlighting concerns regarding the effectiveness and intricacy of existing systems. Zhu et al. (39) compared AI and dentist performance in detecting the mandibular third molar–inferior alveolar nerve relationship, finding that AI and combined dentist + AI approaches improved precision, though not significantly. The analysis suggested that AI-based instruments provided greater detection accuracy and reliability compared to dentists alone, and that the combination of dentist and AI for detection was superior to each method individually. Statistical analysis indicated that the results of the AI-based method and the combined method were not statistically significant or superior to those of dentists-alone interpretation.

By 2025, researchers in Turkey (40) advanced to YOLO11-based tools for predicting M3 extraction difficulty using panoramic radiographs. YOLO11-nano and -medium submodels achieved high precision (97%) and recall (94.6%), offering a robust balance between accuracy and efficiency. Their incorporation into an intuitive graphical user interface facilitates swift evaluation, enhancing surgical planning and patient management. Other frameworks, including VGG16, YOLOv7, and ResNet50, have also demonstrated strong accuracy for M3 detection and classification (41).

Despite these advancements, AI diagnostic systems face limitations. Their accuracy depends heavily on training data quality and cannot yet replace expert interpretation. Ethical issues—privacy, liability, and potential misdiagnosis—necessitate careful oversight (13). ChatGPT-based models, for instance, initially struggled to detect dental infections in radiographs without visual guidance but achieved near-professional accuracy when provided with annotated inputs, illustrating the value of multimodal integration.

A 2024 Saudi review (42) of 21 studies concluded that while AI performs well in assessing M3 morphology and nerve proximity, eruption prediction remains underdeveloped. Similarly, Faadyia et al. (43) emphasized the need for large datasets, standardized diagnostic evaluation, and interdisciplinary collaboration among radiologists, clinicians, and computer scientists to ensure clinically relevant AI development. This process should encompass annotation, segmentation, training, testing, and validation, ensuring that deep learning aligns precisely with the requirements of clinicians in dental practice (43).

When assessing AI software for dental imaging, critical factors include model validation, regulatory approval, system compatibility, supported file formats, reporting functionality, data privacy compliance, cost, and user experience.

In our comparison of three AI-based tools for M3 and M2 pathology, Diagnocat showed the highest accuracy, integrating CBCT and 2D data with clear visual outputs. In comparison, ChatGPT-5 successfully refined its interpretation due to our more specific requirements and the use of graphic markers on the radiograph. Its role should be limited to the semantic processing of textual outputs — including normalization of terminology, generation of structured radiological reports, and assessment of report completeness and coherence. This inclusion reflects the emerging paradigm of multimodal AI integration in radiology, where language models interact with vision models to enhance interpretability, communication, and quality assurance in diagnostic workflows. In contrast, generic radiography tools like x-ray Interpreter produced less reliable results.

AI applications extend beyond diagnostics to surgery. Machine learning models can predict intraoperative complications and nerve injury risk by assessing M3 root proximity to the inferior alveolar nerve on OPGs (44). These preoperative tools improve risk evaluation and surgical planning, though wider adoption will require addressing robustness, security, and liability challenges (45).

### 4.3 Limitations of the study and future perspectives

Limitations of our study derive primarily from its retrospective design and reliance on radiographic data, without incorporating clinical variables or behavioral factors such as oral hygiene, diet, or fluoride exposure. These unmeasured variables may act as confounders influencing second molar pathology, despite efforts to minimize bias by excluding cases with generalized caries or severe periodontal disease. Future prospective studies combining radiographic and clinical data are needed to clarify the relative impact of local vs. systemic factors.

The reliance on two-dimensional radiographic data represents a significant limitation, as orthopantomography does not provide the spatial resolution and anatomical detail achievable with three-dimensional imaging techniques such as CBCT, potentially leading to underestimation or misinterpretation of certain pathologies.

Another primary limitation of this study is the relatively small sample size, which may restrict the generalizability of the findings. While the data provide valuable preliminary insights, the limited number of cases reduces the statistical power and may not fully capture the variability present in broader clinical populations. The modest sample size, while sufficient for proof-of-concept analysis, limits generalizability and statistical power. Future research should therefore focus on larger cohorts (≥300 cases), incorporate multi-observer validation, and include systematic benchmarking of multiple AI tools to enhance diagnostic reliability and support their effective integration into clinical practice. The radiographic evaluation in this study was performed by a single calibrated examiner, ensuring internal consistency in image interpretation. However, the use of a single observer represents a limitation, as it does not allow assessment of interobserver variability. This has been explicitly acknowledged in the manuscript. Future studies should incorporate multiple independent evaluators and include interobserver agreement analysis (e.g., Cohen's kappa) to enhance the reliability and reproducibility of the findings.

The incorporation of AI-assisted tools in this study was exploratory and intended to assess their potential utility within diagnostic workflows. While the technology demonstrated promise in standardizing image interpretation and highlighting regions of interest, its performance remains dependent on algorithmic design, dataset quality, and clinical validation. Accordingly, AI should be regarded as a complementary aid to professional judgment rather than an autonomous diagnostic system. Further research is warranted to establish its reproducibility, accuracy, and practical integration in routine dental practice.

Overall, this research represents an initial step toward integrating multimodal AI in dental diagnostics. By combining image analysis with language-based reporting, the proposed workflow offers a novel framework that enhances consistency and interpretability, warranting further validation in broader clinical contexts.

## 5 Conclusions

This study analyzed the association between impacted mandibular third molars and second molar pathologies, while assessing the feasibility of AI-assisted diagnostics in dental radiology. Findings indicate that impaction type and depth—particularly mesioangular and deeper impactions (Pell & Gregory IIB, IIIB)—are the main determinants of distal caries and periodontal involvement. The identification of M2 distal caries as the most frequent pathology, affecting nearly 60% of cases, and the finding that M2 caries significantly increase the likelihood of M3 caries, provide new insights into the cumulative and interdependent nature of disease patterns in posterior mandibular regions.

Methodologically, the integration of AI-based tools (Diagnocat, x-Ray Interpreter, ChatGPT-5) into a unified radiographic workflow highlights AI's potential to enhance feature detection, reporting consistency, and diagnostic accuracy. Although exploratory due to sample size and algorithmic constraints, the study establishes a proof-of-concept for multimodal AI integration combining visual and language models.

Overall, these findings refine understanding of M3-related risk profiles and demonstrate the value of AI-supported assessment, offering a foundation for future research, clinical standardization, and broader adoption of AI in oral and maxillofacial radiology.

## Data Availability

The raw data supporting the conclusions of this article will be made available by the authors upon reasonable request, in accordance with applicable data protection regulations and institutional requirements for the protection of patient privacy and confidentiality. Requests to access the datasets should be directed to the corresponding author and will be considered provided they comply with ethical and legal restrictions.
